# Subfunctionalization reduces the fitness cost of gene duplication in humans by buffering dosage imbalances

**DOI:** 10.1186/1471-2164-12-604

**Published:** 2011-12-14

**Authors:** Ariel Fernández, Yun-Huei Tzeng, Sze-Bi Hsu

**Affiliations:** 1 Instituto Argentino de Matemática “Alberto P. Calderón”, CONICET (National Research Council of Argentina), Buenos Aires, 1083, Argentina; 2 Department of Computer Science, The University of Chicago, Chicago, IL 60637, USA; 3 Morgridge Institute for Research, Madison, WI 73715, USA; 4 Graduate Institute of Biostatistics, China Medical University, Taichung 40402, Taiwan; 5 Department of Mathematics and National Center for Theoretical Science, National Tsing-Hua University, Hsinchu 300, Taiwan

## Abstract

**Background:**

Driven essentially by random genetic drift, subfunctionalization has been identified as a possible non-adaptive mechanism for the retention of duplicate genes in small-population species, where widespread deleterious mutations are likely to cause complementary loss of subfunctions across gene copies. Through subfunctionalization, duplicates become indispensable to maintain the functional requirements of the ancestral locus. Yet, gene duplication produces a dosage imbalance in the encoded proteins and thus, as investigated in this paper, subfunctionalization must be subject to the selective forces arising from the fitness bottleneck introduced by the duplication event.

**Results:**

We show that, while arising from random drift, subfunctionalization must be inescapably subject to selective forces, since the diversification of expression patterns across paralogs mitigates duplication-related dosage imbalances in the concentrations of encoded proteins. Dosage imbalance effects become paramount when proteins rely on obligatory associations to maintain their structural integrity, and are expected to be weaker when protein complexation is ephemeral or adventitious. To establish the buffering effect of subfunctionalization on selection pressure, we determine the packing quality of encoded proteins, an established indicator of dosage sensitivity, and correlate this parameter with the extent of paralog segregation in humans, using species with larger population -and more efficient selection- as controls.

**Conclusions:**

Recognizing the role of subfunctionalization as a dosage-imbalance buffer in gene duplication events enabled us to reconcile its mechanistic nonadaptive origin with its adaptive role as an enabler of the evolution of genetic redundancy. This constructive role was established in this paper by proving the following assertion: *If subfunctionalization is indeed adaptive, its effect on paralog segregation should scale with the dosage sensitivity of the duplicated genes*. Thus, subfunctionalization becomes adaptive in response to the selection forces arising from the fitness bottleneck imposed by gene duplication.

## Background

A shift in understanding the evolutionary forces that shape the human genome architecture took place when the retention of duplicate genes, a major factor in fostering genome complexity, was recognized to be primarily promoted by random genetic drift [1,2]. Thus, the evolution of genetic redundancy in human and in other higher eukaryotes is enabled by subfunctionalization, a preservation process driven by mildly degenerative mutations that cause complementary loss of subfunctions in different gene copies. These typically dissimilar effects promote the separation of duplicates across cell types or developmental phases, thus making them indispensable to maintain the functional requirements of the ancestral locus. In subfunctionalization, expression-regulatory elements are essentially lost through complementary loss-of-function mutations in paralogs, leading to a partitioning of the function across tissues or developmental phases. Thus, this nonadaptive mechanism is essentially constructive [3], and is enabled by selection inefficiency, which is expected given the small size of the human population [1,3,4].

Yet, as shown in this work, the retention of gene duplicates through subfunctionalization must also encompass adaptive elements. This is so because dosage imbalances arise in the concentrations of the encoded proteins as a result of gene duplication events and the deleterious effects of such imbalances can be mitigated when paralogs are physically separated by subfunctionalization. Dosage imbalances occur when protein concentration levels at specific tissue locations do not fit the stoichiometry of the complexes in which the proteins are involved [5,6]. The complexes may be transient or obligatory with regards to maintaining the structural integrity of the protein. Therefore, dosage sensitivity, that is, the fitness impact of dosage imbalance, must be determined by the extent of functional reliance of the protein on associations [7].

In this work we hypothesize that duplication of dosage-sensitive genes imposes a selection pressure on the fate of the duplicates that is buffered through subfunctionalization. Thus, although originated in random drift, subfunctionalization cannot, and in effect does not, escape the selection forces but rather becomes adaptive to mitigate the fitness bottleneck imposed by the gene duplication event. To validate this hypothesis, we identify a molecular attribute of proteins that is indicative of their dosage sensitivity, thereby quantifying the impact of dosage imbalance effects on the evolution of genetic redundancy. Thus, this work is devoted to prove the following assertion: *If subfunctionalization is indeed adaptive, its effect on paralog segregation should scale with the dosage sensitivity of the duplicated genes*. As shown in this work, this is indeed the case, and in this way, the adaptive nature of subfunctionalization is shown to arise from the imbalance-buffering nature of the process.

Since unicellular organisms lack the buffer of expression diversification, selection pressure on duplicate genes is frequently enough to eliminate one of the duplicates, especially for genes with high dosage sensitivity. Proof of this is the significant decrease in family size with dosage sensitivity encountered in unicellular eukaryotes when compared with higher eukaryotes [7]. Thus, gene duplicates in unicellular organisms are subject to higher purifying selection than their counterparts in multicellular eukaryotes. The scope of this work is to show that subfunctionalization is one of the buffering mechanisms that enable paralog survival in multicellular eukaryotes.

To assess the adaptive contribution to subfunctionalization, it becomes essential to introduce a molecular indicator of dosage sensitivity. As shown in previous work [7], dosage imbalance effects are quantified by *under-wrapping (ν)*, a measure of the packing quality of soluble gene products that determines the extent of reliance of the protein on binding partnerships to maintain its structural integrity [8-12]. Specifically, ν defines in a structure-averaged way the level of hindrance of structure-disruptive backbone hydration. This parameter can be determined directly from protein structure by identifying the percentage of backbone hydrogen bonds (BHBs) that are unburied -the so-called *dehydrons*- and hence poorly protected from competing hydration of the amide and carbonyl [9]. Dehydrons constitute packing deficiencies since they are incompletely "wrapped" by the side-chain nonpolar groups that promote exclusion of surrounding water. Thus, for an individual gene, we get ν = (#dehydrons)/(#BHBs) where the quotient extends over all gene products or encoded proteins. Dehydrons are markers of *compulsory *protein associations that play a structure-protective role by promoting their inter-molecular dehydration [9-12]. Upon protein-protein association, the side-chain nonpolar groups of the binding partner penetrate the microenvironment of the dehydron, contributing to improve its wrapping [12]. This dehydration stabilizes the hydrogen bond in -3.9 kJ/mol [10].

In practice, given the dearth of structurally reported structures when compared with proteome size, dehydrons are often identified from protein sequence using machine-learning methods of inference (Materials and Methods). The rationale for this approach is that, being local indicators of structural disruption, dehydrons belong to a twilight zone between order and disorder that can be identified using a reliable sequence-based predictor of disorder propensity such as PONDR [13].

Recent cross-examination of structural and evolutionary data revealed that duplicates of genes encoding for under-wrapped proteins are exposed to higher deleterious pressure than gene duplicates coding for well-wrapped products. Thus, ν serves as a proxy for dosage sensitivity, as confirmed by a statistically significant negative correlation between family-averaged ν (<ν>) and family size [7].

Paralog survival is dependent on ν with *P *< 10^-17 ^in unicellular organisms, *P *< 10^-6 ^in fly and worm, but *P *< 6.7 × 10^-3 ^in human (Wilcoxon rank test) [7]. This contrast between simple and complex organisms is likely to arise due to the higher complexity of expression regulation in higher eukaryotes. The translation complexity may enable a buffer to dosage imbalance not likely to be found in unicellular organisms. By focusing on evolution-related dosage imbalances, our results corroborate this hypothesis.

The validation of the results asserting the adaptive component of subfunctionalization rests squarely on the legitimacy of under-wrapping as a proxy for dosage sensitivity. Evidence inversely correlating gene family size and under-wrapping [7], evidence arising from analysis of the mechanisms that buffer dosage imbalances in humans [8], and evidence on the reliance of under-wrapped proteins on binding partnerships to maintain their structural integrity [12], all uphold the validity of under-wrapping as a molecular indicator of dosage sensitivity. Nevertheless, a control becomes essential to validate the conclusions of this study. As it turns out, this is the same control that serves to validate the molecular marker adopted [7] and arises from the following rationale: If a specific gene duplication is actually part of a macro-scale event of whole genome duplication (WGD), we expect little or no selection pressure arising from dosage sensitivity since a WGD does not generate a dosage imbalance. Hence, the expression divergence brought about by subfunctionalization of gene duplicates arising from a WGD should result only from random genetic drift, with a minor adaptive contribution. This is indeed the case, as shown in this work.

## Results and discussion

### Adaptive subfunctionalization

We identified the adaptive component of subfunctionalization by determining the extent of paralog segregation through dissimilar mRNA expression as a function of <ν>, normalizing for the divergence time of each family. To support the conjecture of adaptive subfunctionalization in human, we generated an exhaustive database combining genetic, mRNA-expression and wrapping information on human genes and focused on differences in partial degradation of regulatory elements for mRNA-expression across paralogs, a causative of paralog segregation. Only 1957 human gene families from Ensembl Genome Database NCBI36 [14] and reported expression information [15] were found to have coding regions with sustainable ordered structure for free (uncomplexed) subunits. Genes with ORFs coding for disordered regions were excluded from analysis since lack of sustainable structure implies that no wrapping assessment is possible, and structure may only be induced upon association. No paralog protein subunits forming intra-family complexes were found in the database, hence the analysis is free from this confounding factor since obligatory complexation would force coexpression of subunits.

**Figure 1:**
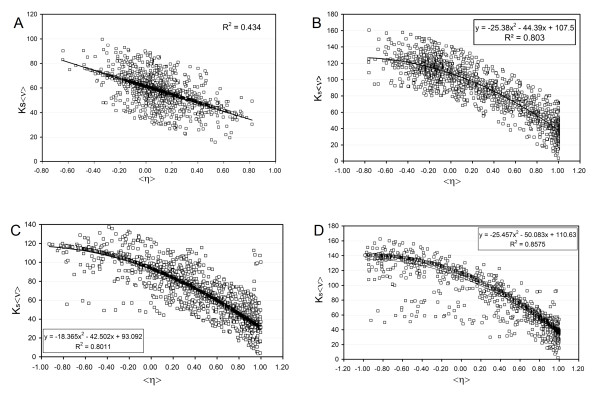
**Paralog segregation buffers dosage imbalance effects and hence scales with dosage sensitivity**. Paralog segregation within a gene family is described by expression correlation parameter <η>, while dosage sensitivity is indicated by <ν>, the average underwrapping of gene products in the family. The <η>-<ν> interdependence is normalized by the divergence time of the family, indicated by Ks. Plot of Ks <ν> versus <η> for 1957 human families (**A**), 1391 fly families (**B**), 2137 worm families (**C**) and 1354 yeast families (**D**) with combined genetic, expression and structural information (Materials and Methods). The correlation coefficient R^2 ^was obtained by regression analysis.

We regarded expression dissimilarities across paralogs as the means of avoiding competing for binding partners upon gene duplication. Three attributes of human gene families were considered: <η>, the mRNA-level expression diversification averaged over paralogs, <ν>, and Ks, the synonymous nucleotide divergence, a proxy for divergence time [16]. For the gene families under consideration we obtain Ks < 2, hence we expect minor saturation effects (cf. [8]). Since paralog divergence is reflective of divergence time, the selection pressure quantified by <ν> is normalized to Ks <ν>, and the buffering effect resulting from subfunctionalization is established by plotting Ks <ν> versus <η> (Figure 1A).

Paralog diversification <η> was estimated by the Pearson coefficient for gene expression vectors corresponding to each paralog pair. For two expression vectors ***X ***and ***Y***, this coefficient is

$$\eta \left(X,Y\right)=\frac{<\left(X-<X>\right)\left(Y-<Y>\right)>}{\sqrt{<X^{2}>-<X{>}^{2}}\sqrt{<Y^{2}>-<Y{>}^{2}}}$$

where *X, Y *are generic coordinates of vectors ***X ***and ***Y ***respectively, and < > in the equation indicates mean over cell types.

Expression diversification in human is more pronounced for genes with high dosage sensitivity in consonance with the hypothesis that subfunctionalization, essentially a nonadaptive process, mitigates dosage imbalance effects. The results (Figure 1A) reveal a significant linear correlation (R^2 ^= 0.43), implying that paralog segregation through subfunctionalization into non-overlapping mRNA-expression patterns becomes enhanced in accord with the dosage sensitivity of the gene duplicates (*P *< 2.2 × 10^-5^). This segregation is needed to avoid dosage imbalances whose effects scale with <ν>.

**Figure 2:**
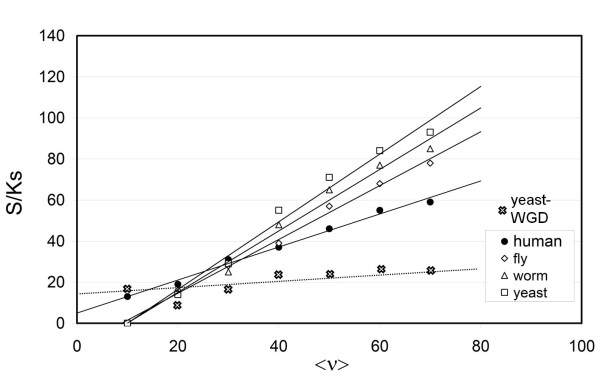
**Paralog segregation sensitivity S as function of dosage sensitivity <ν>**. The results are normalized by divergence-time parameter Ks. Gene families for all four species are grouped by 10% <ν> -ranges and the S/Ks values are averaged over each bin for each species.

Control analyses were carried out for fly (*Drosophila melanogaster*), worm (*Caernohabditis elegans*) and yeast (*Saccharomyces cerevisiae*), for which genetic and expression data distributed across tissue or developmental phases is available and may be combined with disorder-based estimations of <ν> (Materials and methods). Only 1354, 2137, 1391 non-singleton gene families in yeast, worm and fly, respectively, were examined as they have been found to have all coding regions with sustainable ordered structure for free subunits (Additional file 1). The data on these species endowed with higher selection efficiency reveals that paralog segregation becomes more sensitive and more tightly correlated to differences in dosage sensitivity and variations in divergence time, as attested by the quadratic dependence in the Ks <ν>-<η> plot (Figures 1B-D, *P *< 10^-7 ^for fly and worm, *P *< 10^-9 ^for yeast). To contrast the sensitivity of paralog segregation of human relative to control species, we define the family-associated segregation parameter S = 1/2(1- <η>) (0 ≤ S ≤ 1), and plot its Ks-normalized value *versus *<ν> for all four species, grouping families in 10% <ν> -ranges (Figure 2). As expected, paralog-segregation sensitivity increased in the order human < fly ~ worm < yeast, roughly following the species selection efficiency associated with population size [12].

Figure 2 incorporates a control analysis of paralog segregation in a scenario where duplicate genes arise from a whole-genome duplication (WGD) event in yeast [17]. This control is relevant since a WGD does not create a dosage imbalance and hence duplicates arising from a WGD are expected to be subject to little or no selection pressure arising from dosage sensitivity. If our hypothesis is correct as the previous analysis suggests, the expression divergence of duplicates resulting from WGD and brought about by subfunctionalization should result only from random genetic drift, with a minor adaptive contribution. This implies that S should be approximately proportional to the divergence time and independent of <ν>, or S/Ks should remain approximately constant and low relative to the level of segregation experienced by duplications that generate dosage imbalances. This is indeed the case, as shown in Figure 2, leading to the conclusion that in the absence of dosage-related selection pressure, subfunctionalization is indeed the result of random genetic drift (*P *< 4 × 10^-5^), as postulated by Lynch and co-workers [1].

## Conclusions

The preservative role of subfunctionalization in humans and other higher eukaryotes is the result of mildly degenerative mutations likely to cause a differentiating degradation of expression-regulatory elements in gene duplicates. As shown in this work, this process, mechanistically nonadaptive, is subject to the forces of selection and thus develops an adaptive component. This observation motivates the present analysis of the contradictory aspects of constructive neutrality.

Subfunctionalization is nonadaptive insofar as mildly deleterious mutations arise and are fixed in the species population through the vagaries of random genetic drift, and adaptive since subfunctionalization becomes also a buffer of the dosage imbalances that arise from gene duplication. When compared with species with higher selection efficiency, paralog segregation in human is not nearly as complete or efficient for families with high dosage sensitivity. Yet, the results from Figures 1A and 2 reveal a significant adaptive role of human subfunctionalization when regarded as a buffer of the effects of dosage imbalance quantified by the gene dosage sensitivity. Thus, the statistical analysis presented in this work unravels the fact that a process that is mechanistically nonadaptive when viewed as enabler of duplicate retention may have adaptive consequences since it also serves to mitigate the selection pressure arising from duplication events.

This picture is further validated by examining a scenario in which gene duplications do not generate dosage imbalances. Such is the case with whole genome duplication (WGD) [17]. In this case, we expect and corroborate that the dominant evolutionary force leading to paralog segregation through subfunctionalization is random genetic drift.

The mechanistic effects of population size on the efficacy of subfunctionalization were emphasized by Lynch [1], and are clearly confirmed in our study (Figures 1, 2). To further test this dependence, it would be desirable to contrast paralog segregation in endosymbionts versus the segregation undergone by the orthologs of these paralogs in the free species. However, the expression of genes in an endosymbiont is highly coordinated and correlated with gene expression in the host [18], thereby masking the effects of population size on paralog segregation.

## Methods

Gene information was obtained from the following sources: *Saccharomyces cerevisiae (strain S288C)*, Saccharomyces Genome Database http://www.yeastgenome.org/ (SGD1.01); *Caenorhabditis elegans*, WormBase http://www.wormbase.org/ (WB170); *Drosophila melanogaster*, Berkeley Drosophila Genome Project http://www.fruitfly.org/ (BDGP 4.3); *Homo sapiens*, Ensembl Genome Database (NCBI36). Using the Ensembl gene family annotation [14], 6,024 yeast genes were grouped into 4,661 families, 20,173 worm genes were grouped into 11,503 families, 14,116 fly genes were grouped into 9,477 families, and 22,357 human genes were grouped into 12,394 families.

Gene expression data for different species were obtained from different sources: Novartis Gene Expression Atlas [15] for human, FlyAtlas for fly [19], PUMAdb for worm [20], *Saccharomyces *Genome Database for yeast [21]. For human, the gene expression dataset contains expression levels across a panel of 73 normal human tissues (samples of the 6 cancer-related tissues were not included). The PUMAdb dataset contains gene expression levels for worm at 6 different developmental time points (egg, L1, L2, L3, L4, and young adult) in two different strains (N2 and CB4856). The *Saccharomyces *Genome Database contains yeast mRNA expression levels during the 5 metabolic adaptation phases representing the transition from glucose-fermentative to glycerol-based respiratory growth. Paralogous genes arising from yeast WGD were obtained from Kellis et al. [22].

Synonymous nucleotide divergence, Ks, across paralog pairs was determined using the PAML package [23]. Its relevance as a surrogate for divergence time in a gene family is clearly delineated in [16].

The wrapping of a backbone hydrogen bond, ζ, was computed directly from PDB structural coordinates for gene products whenever available [8,11]. This local parameter is computed by determining the number of side-chain nonpolar groups contained within a desolvation domain around the bond. This domain was defined as two intersecting spheres of fixed radius (~thickness of three water layers) centered at the α-carbons of the residues paired by the hydrogen bond. In structures of soluble proteins, backbone hydrogen bonds are protected on average by ζ = 26.6 ± 7.5 nonpolar groups for a desolvation sphere radius 6Å. Dehydrons lie in the tails of the distribution, i.e. their microenvironment contains 19 or fewer nonpolar groups (ζ ≤ 19), so their ζ-value is below the mean minus one standard deviation.

The parameter ν can be determined from protein sequence, an imperative given the scarcity of structural information relative to proteome sizes. Since they represent structural vulnerabilities, dehydrons belong to a twilight zone between order and disorder [12]. This characterization is suggested by a strong correlation between two local parameters: wrapping (ζ), giving the number of protective nonpolar groups around the BHB, and propensity for structural disorder (f_d_) [11,12]. The correlation reflects the fact that the propensity for backbone hydration is indicative of a propensity for structure disruption. The parameter f_d _is a sequence-based score generated by the program PONDR-VLXT [24], a predictor of native disorder that takes into account residue attributes and their distribution within the window interrogated [13]. The disorder score (0 ≤ f_d _≤ 1) is assigned to each residue within a sliding window, representing the predicted propensity of the residue to be in a disordered region (f_d _= 1, certainty of disorder; f_d _= 0, certainty of order). The strong correlation between the disorder score of a residue and wrapping of the hydrogen bond engaging the residue (if any) provides a sequence-based method of inference of dehydrons and supports the picture that such bonds belong to an order-disorder twilight zone. Thus, dehydrons can be inferred in regions where the disorder score lies in the range 0.35 ≤ f_d _< 0.95, which corresponds to a marginal BHB wrapping with 7 ≤ ζ ≤ 19.

## Abbreviations

BHB: backbone hydrogen bond; PUMAdb: Princeton University MicroArray database.

## Competing interests

The authors declare that they have no competing interests.

## Authors' contributions

AF conceived the work, developed the theoretical framework, collected the data and wrote the paper. YHT analyzed the data. SBH critically assessed the theoretical concept, contributed to the model. All authors read and approved the final manuscript.

## Supplementary Material

Additional file 1 **Non-singleton gene families in yeast, worm, fly and human with coding regions able to sustain free subunits with ordered structure**. Click here for file
